# Diphtheria

**Published:** 1880-12

**Authors:** 


### DIPHTHERIA.

Diphtheria is generated by breathing impure air, such as comes from damp apartments, dirty cellars, gutters, sinks, decaying matters, pools of standing liquids and other sources of filth. It is usually confined to persons from two to fifteen years of age. The atmosphere, the breathing of which causes the disease, seems to be full of living things, vegetable and animal, the bacteria and micocoppins, some of which lodge in the throat and form white splotches, which are distinctive of the malady, and whose presence quickly poisons the blood ; hence there should not be a moment's delay in sending for a physician, as the march of the disease is always rapid and its virulence increases every hour.

As with most other diseases, diphtheria is more likely to attack those whose systems have been debilitated by illness, poor diet, or any cause whatever. Any irritation of the throat prepares the way for the disease. Any person affected should be taken to an upper room, into which no one should come but those in perfect health, and who have not the slightest scratch or sore on any part of the body, particularly the hands. The room should be ventilated all the time, all discharges should be quickly removed, the clothing frequently changed and at once covered with water containing carbolic acid.

Diphtheria taken from another person is more malignant than when generated by bad air.

### TREATMENT OF DIPHTHERIA.

Take from two to five grains of chlorate of potash, put it far back on the tongue, allow it to melt gradually, and repeat every hour until a decided improvement takes place, which is usually in a few hours. One of the best plans of treatment is the following :

#### GARGLE.

| | |
|---|---|
| Chlorate of potassa | 2 drachms. |
| Hot water | 6 ounces. |
| Alcohol | 4 drachms. |
| Creosote | 8 drops. |
| Muriatic acid | 30 drops. |

This is to be used as a gargle every thirty minutes.

#### INTERNALLY USE THE FOLLOWING REMEDY :

| | |
|---|---|
| Chlorate of potassa | 3 drachms. |
| Water | 6 ounces. |
| Sugar | 1 ounce. |
| Tincture of muriate of iron | 2 drachms. |

DOSE.—A teaspoonful every four hours.

It is claimed that the above treatment will cure nineteen cases out of twenty.

The principal point is to find out what will destroy the bacteria.

Tannin will kill them in two hours. A solution of copperas, that is, sulphate of iron, will kill them; also chlorine water and dilute muriatic, sulphuric and nitric acids. If copperas is used as a gargle, it should not be used stronger than a piece half as large as a nutmeg, dissolved in a pint of water; or a level teaspoonful of tannin dissolved in a teaspoonful of warm water; but it is better to rely on chlorine washes above named; and it would be a safe plan, should any member of a family have diphtheria, for each of the others to gargle the mouth every hour with chlorine solution.

Instead of using solutions, a few grains of either chlorate of potash, tannic acid or copperas, known as sulphate of iron, may be placed dry, far back on the tongue, to dissolve gradually and spread over the surfaces. It is a more simple method of applying these remedies, and would be more likely to reach farther down the throat and to remain longer in contact with the diseased surfaces than when applied in gargles or swallowed.

THESE REMEDIES ARE POWERFUL TO CURE IN PROPORTION TO THE PROMPTNESS WITH WHICH THEY ARE USED.

### EGYPTIAN PAD.

Gum galbanum, ground, one-half ounce; gum myrrh, ground one-quarter ounce; gum camphor, ground, one drachm; gum ammoniac, ground, one-half ounce; gum benzoin, ground, one-quarter ounce; sandalwood, ground, two drachms. Mix them. If the gums are too moist to admit of reduction, dry them with the aid of heat. The quantity given is for one pad. It should be worn over the sternum, about midway between the heart and liver. These pads are said to prevent and cure fever and ague and all malarious diseases.—*Copied from Nelson's "Hand Book of Private Formulas."*

### TOOTHACHE DROPS.

Powdered gum camphor, one ounce; chloral hydrate, one ounce. Rub them together in a wedgewood mortar until they liquify. Apply to the cavity on a small piece of cotton.—"*Hand Book of Private Formulas.*"

